# Effectiveness of a community-based support programme to reduce social inequality in exclusive breastfeeding: study protocol for a cluster-randomised trial

**DOI:** 10.1186/s12889-023-15256-z

**Published:** 2023-03-08

**Authors:** Henriette Knold Rossau, Ingrid Maria Susanne Nilsson, Marianne Busck-Rasmussen, Claus Thorn Ekstrøm, Anne Kristine Gadeberg, Jonas Cuzulan Hirani, Katrine Strandberg-Larsen, Sarah Fredsted Villadsen

**Affiliations:** 1 Section of Social Medicine, Department of Public Health, University of Copenhagen, Øster Farimagsgade 5, 1353 Copenhagen, Denmark; 2 The Danish Committee for Health Education, Classensgade 71, 5th floor, 2100 Copenhagen, Denmark; 3 Section of Biostatistics, Department of Public Health, University of Copenhagen, Øster Farimagsgade 5, 1353 Copenhagen, Denmark; 4 The Danish Center for Social Science Research, Herluf Trolles Gade 11, 1052 Copenhagen, Denmark; 5 Section of Epidemiology, Department of Public Health, University of Copenhagen, Øster Farimagsgade 5, 1353 Copenhagen, Denmark

**Keywords:** Breast feeding, Community health, Complex interventions, Cross-sectoral consistency, Delivery of health care, Health care sector, Health visitor, Postnatal care, Randomized controlled trials, Socioeconomic factors

## Abstract

**Background:**

Breastmilk is the ideal nutrition for infants, and breastfeeding protects infants and mothers from a range of adverse health outcomes. In Denmark, most mothers initiate breastfeeding but many cease within the first months resulting in just 14% reaching the World Health Organization recommendation of six months of exclusive breastfeeding. Furthermore, the low breastfeeding proportion at six months is characterised by a marked social inequality. A previous intervention tested in a hospital setting succeeded in increasing the proportion of mothers breastfeeding exclusively at six months. However, most breastfeeding support is provided within the Danish municipality-based health visiting programme. Therefore, the intervention was adapted to fit the health visiting programme and implemented in 21 Danish municipalities. This article reports the study protocol, which will be used to evaluate the adapted intervention.

**Methods:**

The intervention is tested in a cluster-randomised trial at the municipal level. A comprehensive evaluation approach is taken. The effectiveness of the intervention will be evaluated using survey and register data. Primary outcomes are the proportion of women who breastfeed exclusively at four months postpartum and duration of exclusive breastfeeding measured as a continuous outcome. A process evaluation will be completed to evaluate the implementation of the intervention; a realist evaluation will provide an understanding of the mechanisms of change characterising the intervention. Finally, a health economic evaluation will assess the cost-effectiveness and cost-utility of this complex intervention.

**Discussion:**

This study protocol reports on the design and evaluation of the Breastfeeding Trial – a cluster-randomised trial implemented within the Danish Municipal Health Visiting Programme from April 2022 to October 2023. The purpose of the programme is to streamline breastfeeding support provided across healthcare sectors. The evaluation approach is comprehensive using a multitude of data to analyse the effect of the intervention and inform future efforts to improve breastfeeding for all.

**Trial registration:**

Prospectively registered with Clinical Trials NCT05311631 https://clinicaltrials.gov/ct2/show/NCT05311631

**Supplementary Information:**

The online version contains supplementary material available at 10.1186/s12889-023-15256-z.

## Background

Breast milk is the ideal nutrition for infants. A meta-analysis has demonstrated that breastfeeding protects the infant against infections and malocclusion, increases intelligence and potentially reduces overweight and diabetes later in life [1]. Furthermore, breastfeeding protects the mother against breast cancer and possibly also against ovarian cancer and type 2 diabetes [1]. Thus, few health behaviours have as important public health benefits as breastfeeding given its potential to improve life chances, health and well-being [2]. The World Health Organization (WHO) recommends exclusive breastfeeding of infants in the first six months of life when possible, and partial breastfeeding alongside complementary foods for up to two years of age or beyond [3].

The proportion of women who initiate breastfeeding in Denmark is among the highest in the world [4] with more than 97% initiating breastfeeding [4, 5]. Yet, in recent years, the proportion of Danish women breastfeeding exclusively for six months has reached no more than 14% [6]. In line with the WHO, the Danish Health Authority recommends six months of exclusive breastfeeding, simultaneously recommending that solids is not introduced to the infant’s food before the age of four months [7]. Consequently, Danish exclusive breastfeeding rates drop markedly around four months after birth [8].

A social gradient in breastfeeding has been documented in high-income countries [4, 9, 10] including Denmark [11]. Young mothers and mothers with a low socioeconomic position (SEP) are the least likely to initiate breastfeeding and most likely to cease breastfeeding before six months [12, 13]. However, a recent study found that after adjusting for psychosocial factors such as breastfeeding self-efficacy and sense-of-security, education no longer had a prognostic effect on the duration of exclusive breastfeeding [14], implying that efforts to underpin self-efficacy and sense-of-security may reduce the social gradient. Previous research has found that early breastfeeding cessation is frequently due to a perceived insufficient milk supply [15–17] and that mothers at risk of early breastfeeding cessation require more support from healthcare providers to achieve their breastfeeding goals [2]. Consistency in breastfeeding support information is important for mothers in general and for young mothers in particular [18]. Furthermore, young mothers frequently require more specific support in the form of affirmation and recognition of their efforts, which boosts their self-efficacy and motivation [18–22]. The timing and frequency of the support given are important, but evidence is insufficient to recommend a specific schedule [23].

In Denmark, breastfeeding support is initiated immediately after giving birth in the maternity ward setting. After hospital discharge, breastfeeding support is continued in the families’ homes within the context of a universal, municipality-based and tax-financed health visiting programme [24]. Danish health visitors are registered nurses with a minimum of three years of full-time clinical experience and an additional 1.5 years of training [25]. With early discharge following birth, most breastfeeding support is provided by the health visitors. Challenges relating to inconsistency in breastfeeding recommendations and cross-sector breastfeeding support have been experienced by women [26].

In 2013–14, Nilsson et al. (2017) implemented a breastfeeding support programme in a hospital setting for mother-infant dyads facing early discharge [27]. When compared with usual care, the intervention improved exclusive breastfeeding at six months postpartum and reduced infants’ readmissions to hospital within the first week of life [27]. The intervention included training of healthcare professionals in providing improved breastfeeding support, aiming to increase mothers’ breastfeeding self-efficacy by simplifying breastfeeding counselling to four key principles: 1) Skin-to-skin contact, 2) Frequent breastfeeding, 3) Proper positioning and 4) Joint parenting task [27]. Subsequently, these principles were included in the breastfeeding recommendations of the Danish Health Authority [7] and either partially or completely implemented throughout Danish maternity wards.

Knowing that the previous intervention was effective, that cross-sector breastfeeding inconsistencies exist, that most breastfeeding support is provided following discharge with an estimated 97% of parents accepting the health visiting programme [28] and that a social gradient in breastfeeding exists, we decided to develop the ‘Breastfeeding – A Good Start Together’ trial (henceforth termed The Breastfeeding Trial). Our ambition was to strengthen breastfeeding support offered within the municipality-based health visiting programme and to align it with the dominating current practice in the hospital setting. The aim of the intervention was to raise exclusive breastfeeding rates and reduce social inequality in breastfeeding.

Prior to adapting the intervention from use in a hospital setting to use in the health visiting programme, we identified important knowledge gaps. A narrative literature review revealed that mothers who were young or had a low educational attainment frequently wished and intended to breastfeed but found themselves overwhelmed by their birth, the transition into motherhood and various breastfeeding challenges. These complexities meant that they had a lower threshold for giving up breastfeeding [29]. They also struggled with embarrassment related to breastfeeding and were easily influenced by attitudes in and experiences with breastfeeding in their social networks [29]. A qualitative study identified the early period after discharge as an especially vulnerable period. During this period, e.g., the stability, self-efficacy, sense of stigma towards age and breastfeeding attitude in families would potentially make them opt out of breastfeeding [30]. The families experienced a need for basic, practical knowledge and visual information; they found such information online or purchased help from private lactation consultants. However, a positive relationship with the health visitor was highlighted as essential to parents’ benefit from breastfeeding support [30].

As social inequality in breastfeeding may contribute to persistent health inequities across generations [31], interventions to increase breastfeeding are an important public health matter. Previous research call for future breastfeeding interventions exploring how support may best be provided consistently, and describe in detail the attributes of the intervention, standard care and the population group studied to provide strong evidence for future reference [2]. The goal of this study protocol is to describe the Breastfeeding Trial and the standard care provided, while outlining plans and methods for analysing effects and describing other pertinent evaluation techniques.

### Objectives

The primary objective of the Breastfeeding Trial is to assess the effect of a community-based breastfeeding support programme delivered by health visitors in primary healthcare on the duration of exclusive breastfeeding and social inequality in breastfeeding among women in Denmark. Our hypothesis is that women who receive strengthened breastfeeding support in conjunction with the breastfeeding support offered in the hospital setting will overcome breastfeeding challenges and therefore breastfeed exclusively for a longer period of time. We anticipate that by administering a higher dose of the intervention to mothers who are young or have a low educational attainment, the effects will be even stronger in this group, thereby reducing social inequality in breastfeeding.

We will conduct a comprehensive, multidisciplinary evaluation of the trial by investigating whether the present complex intervention is effective compared with usual breastfeeding support, with respect to:

- The proportion of women who are breastfeeding exclusively at four months postpartum
- The overall duration of exclusive breastfeeding
- Reducing social inequality in breastfeeding

In a process evaluation, we investigate the processes of implementation, specifically:

- Facilitators of and barriers to implementing the intervention
- Dose, delivery and fidelity of the intervention
- How the training programme affects health visitors’ knowledge, self-efficacy and action competencies regarding breastfeeding support

Using a realist evaluation approach, we investigate the relationship between context, mechanisms and outcomes to determine *whether* and *how* the intervention works. The realist evaluation will focus primarily on families in which the mother is young or holds a low educational attainment (i.e., the group that will receive an intensified version of the intervention (see a description of the intervention in Table 2)). More specifically, we will:

- Investigate important mechanisms of change by examining families and health visitors’ response to and interaction with the intervention
- Analyse how contextual factors promote or inhibit the implementation process and the expected mechanisms of change and the potential consequences
- Investigate how the intervention works for mothers who are at an increased risk of early breastfeeding cessation

Finally, in a health economic evaluation, we will assess:

- The cost-effectiveness of the intervention
- The cost-utility of the intervention

### Definitions

In this trial, exclusive breastfeeding means that the infant exclusively receives breast milk after discharge from the hospital, regardless of feeding mode. In Denmark, and hence in this trial, exclusive breastfeeding allows for supplementing with, e.g., water and/or a maximum of one meal of infant formula per week [7]. Partial breastfeeding means that, in addition to breast milk, the baby receives infant formula or other diet elements several times a week or daily.

## Methods and analysis

### Study design

The Breastfeeding Trial is a cluster-randomised trial with two arms: an intervention and a control arm. As the developed programme constitutes a complex intervention, we adhere to the Medical Research Council (MRC) framework and wish to combine the effectiveness perspective with a theory-driven evaluation, analysing how the activities generate effects to provide comprehensive evidence for decision making [32].

We use survey data to reveal any effect on the outcome of exclusive breastfeeding at four months postpartum. Consequently, this study protocol reports on the survey study in accordance with the SPIRIT Statement [33]. Additionally, a register-based difference-in-difference study and a process-, realist- and health economic evaluation will be conducted as part of the trial. We have included information about these methodologies in the current study protocol. The integration and design of the various aspects of investigation are guided by a programme theory that depicts in a logic model frame how the activities of the intervention are expected to produce outcomes (see Fig. 2).

We analyse the effect of the intervention between the trial arms by harvesting data from continuous monitoring of breastfeeding duration in nation-wide registries [34]. In a process evaluation [35], we assess the fidelity and quality of the implementation of the intervention (dose, adaptations and reach) and identify contextual factors potentially associated with variation in outcomes. Furthermore, the intervention mechanisms will be analysed using a realist approach [36, 37] in the intervention arm to understand how intervention activities and resources in the specific contexts potentially generate mechanisms and outcomes, with special focus on families with a low SEP. Finally, a health economic assessment will be conducted using cost-effectiveness and cost-utility approaches [38].

### Study population

**Fig. 1:**
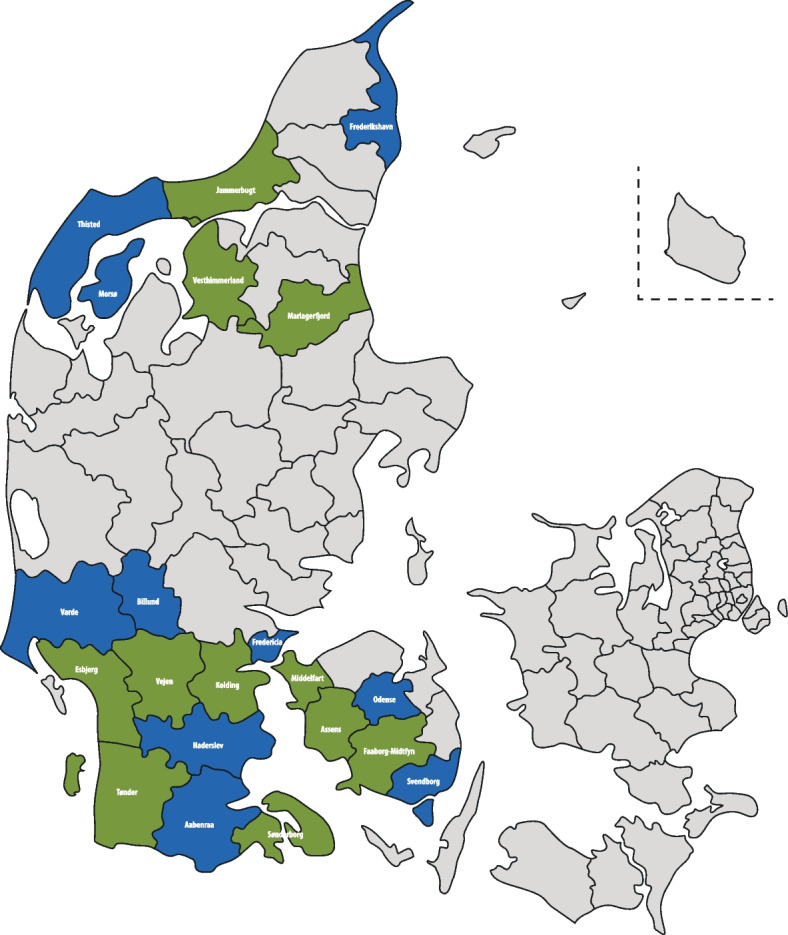
Map of Denmark. Green areas indicate intervention clusters; blue areas, control clusters

The study setting includes 21 municipalities in Denmark, all of which are situated in the North Denmark Region (*n* = 6) or Region of Southern Denmark (*n* = 15) (see Fig. 1). Denmark consists of a total of five regions. The two selected regions have some of the highest proportions of mothers who are of young age and/or have a low educational attainment. Each of the 21 participating municipalities constitutes a cluster in the trial.

#### Eligibility criteria

All women giving birth in the 21 municipalities during the project period from April 2022 to end of 2023 are eligible for inclusion in the trial provided they are: 1) ≥ 15 years old, 2) intend to breastfeed their infant, 3) were discharged from the hospital less than seven days after giving birth^1^, 4) gave birth to a singleton, and 5) had a term born child. The exclusion criteria are: 1) insufficient Danish and English language skills to respond to questionnaires and understand the support conveyed in either Danish or English, 2) known substance use disorder or use of illegal drugs, and/or 3) known morbidity or disablement affecting their ability to breastfeed. Fathers/partners are invited to participate based on their partner’s eligibility and must be able to understand Danish or English.

### Description of the intervention

#### Control group – care as usual

New families in the control clusters will receive standard care breastfeeding support. The Danish Health Authority provides guidance on what should be provided as standard care. Home visits are offered to all children and their families from birth to two years of age. Standard care is a minimum of five visits during the infant’s first year of life for a normal trajectory, with an option for provision of needs-based visits [28]. The aim of standard care is prevention and health promotion where relevant topics are covered depending on the timing of the visits [28]. Table 1 presents a description of standard care.

**Table 1:** Description of standard care

**Visits**
The recommendation from the Danish Health Authority is that a minimum of five consultations (mostly home visits) are offered to all children and their families from birth to one year of age under the auspices of the municipality-based health visiting programme [28]:
1. First visit within the first week of life (for mothers discharged < 72 h postpartum)
2. Second visit during the first month of life
3. Third visit when the infant is two months old
4. Fourth visit when the infant is four to six months, and
5. Fifth visit when the infant is eight to ten months old
In the standard care lies an option for health visitors to offer families so called ‘needs based visits’ or follow-up if the health visitor considers this required [28]
**Content of the visits**
The content of the standard care is prevention and health promotion, and subjects depend on the timing of the visits [28] The topics include:
• Breastfeeding support and –cessation prevention
• Infant thriving
• Family formation
• Physical and mental condition of the infant, including infant-parent attachment
• Infant self-regulation
• Psychomotor development
• Parents’ mental well-being (including screening for postpartum depression)
• Infants’ eating- and sleeping patterns
• Introduction to solid foods (4–6-month visit)
• Language development, and
• Prevention of accidents

#### Intervention group

In the intervention arm, the intervention is implemented into the already existing health visiting programme. All new parents in the intervention clusters will thus receive strengthened breastfeeding support if they accept the health visiting programme. The four key principles from the original 2013–2014 intervention at hospitals are adapted to the municipality setting. Consequently, the current intervention seeks to strengthen consistency in breastfeeding support across sectors and thus to reduce any confusion among parents. The core of the intervention is building a trustful relation between health visitors and families and that the health visitors strengthen parents’ action competency. The intervention builds on theories of tailored communication and support [39, 40] and breastfeeding self-efficacy [41, 42]. The activities generating strengthened support are competence training for health visitors and development of new health education materials targeting the parents. The provision of pregnancy visits will underpin trustful relationship building between the family and the health visitor. The duration and form of the training and the contents of the intervention are presented in more detail in Table 2.

**Table 2:** Description of the intervention programme

**Development and implementation**
For the adaptation of the previous successful breastfeeding intervention done by Nilsson and colleagues (2017) in hospital setting, an assessment of the needs of families following discharge from hospital after birth and until six months postpartum was carried out. Programme objectives corresponding to findings from the needs assessment formed the basis of the adaptation. In the adaptation process, a participatory approach including health visitor representatives from each of the intervention municipalities was chosen to enhance ownership, to match the organisational structure of the setting (the Danish Health visiting programme), and to ensure implementation in the intervention municipalities. The final intervention programme consisted of a breastfeeding programme for parents and a related training programme for health visitors. In December 2021, 210 health visitors from the 11 intervention municipalities completed a preparatory 3.5-h e-learning course, and in March 2022 they participated in the 15-h physical attendance interactive training programme supported by a written manual. Following training, the health visitors immediately initiated implementation of the programme in their municipalities
**Topics in the training programme for health visitors**
• Current evidence-based knowledge about breastfeeding, including:
- Anatomy of the breast and physiology of lactation
- Preparing for breastfeeding
- Breastfeeding positions
- Suckling technique
- Alleviating breastfeeding problems, for example by strengthening parents’ action competence
- Breastfeeding as a joint parenting task
- The social context of the parents and its’ influence on breastfeeding
• Tailoring communication
• Breastfeeding self-efficacy – how to improve and support parents’ self-efficacy
**Objective of the parental breastfeeding programme**
To support parents who want to breastfeed in establishing effective breastfeeding by improving health visitors’ and parents' breastfeeding knowledge, and enhancing parents’ self-efficacy, and action competency through a focus on four simple key principles and tailored information
**Content of the parental breastfeeding programme**
The programme consists of four key principles:
• Skin-to-skin contact as much as possible during the first week while the mother and father are awake [43]
• Frequent breastfeeding defined as a minimum of eight times including identifying the infant’s cues for being ready for breastfeeding and signs of getting enough milk [44, 45]
• Proper positioning of the mother-infant dyad including introducing the parents to laid-back breastfeeding and focus on the mothers’ experience of pain and relaxation as guidelines for positional changes [46, 47]
• Acknowledgment of the mother and the father as equal parents with different roles in relation to breastfeeding [48]
The parents’ breastfeeding self-efficacy and breastfeeding knowledge is intended to be supported by the communication based on Bandura’s theory of self-efficacy and the related four sources to increase self-efficacy: 1) Mastery experiences, 2) Vicarious experience, 3) Social persuasion, and 4) Emotional arousal [42]. Kreuter’s theory of tailoring knowledge to the specific needs of the individual [49] is furthermore included to address findings from the needs assessment [30]
Supportive health educational materials include:
• A dialogue sheet
• A pamphlet with visuals for the changes in colour of a healthy infants’ stool during the first week
• A postcard with the four key principles
• A website with written knowledge, videos, podcasts, a quiz about breastfeeding aimed at preparing parents for breastfeeding, and furthermore a trouble shooting guide

Should families decline further visits by the health visitor, they will no longer receive the intervention. The investigators will make no efforts to retain the families in the study.

##### Intensified intervention

In the intervention arm, families in which the mothers are younger than 25 years when they give birth (young age) and/or have primary school or vocational training as their highest educational attainment (low education) are offered an intensified intervention following the same breastfeeding principles, but they will receive a higher dose of the intervention. The aim is for health visitors to be proactive in delivering support and preventing breastfeeding cessation in this group, which carries an increased risk of early cessation. The intensified intervention is delivered within the first five months through additional follow-up phone calls (*n* = 7) and a single extra visit, all of which are scheduled to occur in-between the home visits (see details in Additional File 1). If a mother receiving follow-up by telephone ceases breastfeeding, she will no longer receive the telephone follow-up. Families accepting the intensified intervention are free to decline further extra telephone calls and/or visits.

The intervention is expected to reach > 6,000 families in the trial period, of whom 30–40% are expected to be in the target group for the intensified intervention. After the trial period, health visitors in the control clusters will receive the training and supportive materials developed during the intervention.

**Fig. 2:**
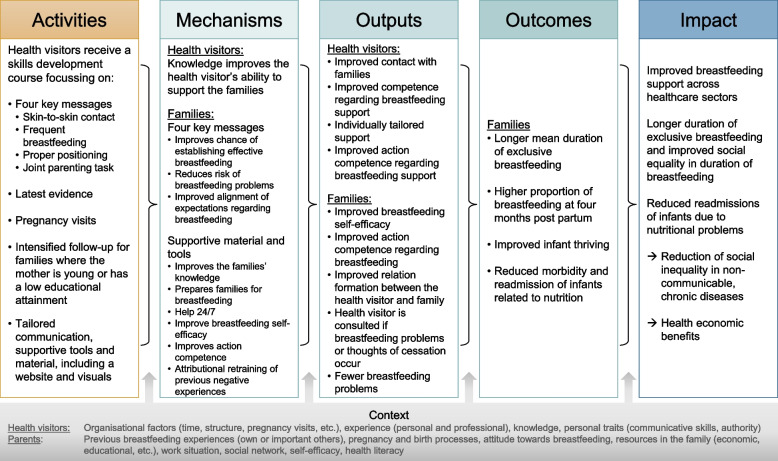
Programme theory of the Breastfeeding Trial

Figure 2 presents the programme theory in a logic model structure. The programme theory guides evaluation by outlining our hypothesis as to which mechanisms drive intended changes in outputs and outcomes to be measured, e.g., the hypothesis that the breastfeeding support programme will promote frequent breastfeeding, which will increase the likelihood of establishing effective breastfeeding, among others because milk production is stimulated causing sufficient milk to reach the infant, which, in turn, is hypothesised to lower the risk of readmissions.

#### Randomisation

The participating municipalities were randomised for either the intervention or control arm by a statistician using computer-generated cluster randomisation. Randomisation was stratified by Danish region and number of annual births per municipality (0 =  < 900, 1 = 900 +). The number of annual births in a municipality was associated with maternal educational level and age as urban cities with more inhabitants and thus more births had fewer mothers with a low educational level and young age than rural districts with fewer births. Stratification of randomisation was performed to increase the exchangeability of the two trial arms.

The intervention works by strengthening competencies at health visitor level. Once achieved, such competencies are difficult and unethical not to use, wherefore randomisation at the level of families was discarded. Randomising at the cluster level serves to mitigate the risk of contamination between trial arms. Allocation to the intervention or the control arm is based on address and municipal affiliation.

### Outcome measures of the effectiveness trial

Data on the primary outcome measure of exclusive breastfeeding at four months are collected through a survey. To gain as much insight into the intervention mechanisms and effects as possible, we collect data from various sources as described in the aim. As the trial was designed to show effects on survey-based measures, we elaborate on the survey trial design below and afterwards describe the supplementary data sources.

### Survey data

**Fig. 3:**
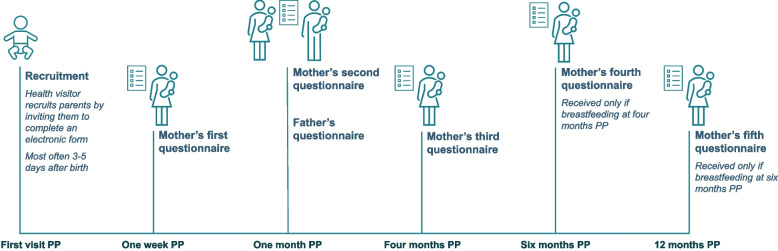
Overview of the survey study questionnaire distribution

The primary outcome measure is studied by using self-reported measures collected via self-administered web-based questionnaires. Survey questionnaires are distributed via emails to mothers recruited via the health visiting programme at the following five postpartum time-points: one week, one month, four months, six months and 12 months. Additionally, fathers/partners were invited to a survey at one month postpartum (see Fig. 3 for an overview timeline). From the surveys, we also study a range of secondary outcome measures.

#### Primary outcome measure

1. Proportion of mothers who exclusively breastfeed at four months postpartum.

#### Secondary outcome measures

The secondary objectives are to assess the effectiveness of the breastfeeding support programme on:

1. Duration of exclusive breastfeeding (continuous)
2. Infant readmissions to hospital related to nutrition
3. Mothers’ experiences of breastfeeding problems
4. Parental trust in the health visitor
5. Maternal breastfeeding self-efficacy
6. Paternal feeling of inclusion in the breastfeeding support
7. Breastfeeding in mothers with overweight

### Sample size and power calculations

For sample size calculation, an estimated eight clusters in each arm were used, totalling 16 clusters. Expected participation rate was 80% with 30% attrition from the first questionnaire at one week postpartum to the third questionnaire at four months postpartum. A power calculation was made using breastfeeding frequencies from 2016 made available from The National Child Health Register [50]. The ambition was to raise the proportion of women breastfeeding at four months in the intervention clusters to the national level. In the design phase, we obtained data on the mean prevalence of breastfeeding at four months in the project regions (49%) and at national levels (56%) in 2016 from the National Child Health Register. The observed difference in the proportion of women exclusively breastfeeding for a minimum of four months corresponds to an odds ratio of 1.32. In the power calculations, an interclass correlation coefficient (ICC) of 0.001, as observed in the previous intervention study [27], was used. Reaching a statistical power of 80% with a 5% significance level is thus achievable provided data are collected from 111 mothers in each cluster. In each cluster, the sample size was extended to ensure that a minimum of 52 mothers were young or had low educational attainment. This yields a power of 80% to investigate an increase in exclusive breastfeeding at four months from 45 to 55% in this subgroup.

### Recruitment to the survey

The health visitors will recruit the families during their first visit after birth. Health visitors have been instructed to inform about the project verbally and in writing in families where the mother meets the eligibility criteria (see information sheet in Appendix A). Enrolment is done by the health visitors who collect families’ consent and help fill in the families’ information in a secure electronic recruitment form. Furthermore, the health visitor assesses whether the mothers qualify for the intensified intervention based on her age and educational level. Participation in the survey and the intensified intervention are independent, i.e., mothers declining participation in the survey may still accept the intensified intervention.

Municipalities will stop recruiting when the required number of mothers with data from the first three questionnaires has been reached, as outlined in the description of the sample size calculation.

### Data collection

The data collection period commenced on 26 April 2022, in both trial arms. From mid-June it has been possible to assess the full intervention in the intervention clusters, including monitoring the effect achieved by introducing breastfeeding counselling in the pregnancy visit. Local variations will exist in the duration of data collection due to a varying number of births in the clusters. Data collection is projected to end by October 2023 in the municipalities with fewest births.

The questionnaires are distributed to families via personal emails and followed up by two reminders sent by text message (SMS).

Mothers for whom reading and filling out a questionnaire are very challenging are offered a phone call or Microsoft Teams call by a project student assistant who will help them complete the questionnaire. At recruitment, the mother will decide whether to accept or decline this offer.

An overview of the data collection timeline is given in Fig. 3, and Table 3 shows which outcomes and covariates are collected from each survey wave.

**Table 3:** Overview of quantitative data collection, source and timepoints

Outcome measures	Survey data						Register data
*Sources*	**Mother**					Partner	
Time point since birth (PP)	One week	One month	Four months	Six months	Twelve months	One month	
**Primary**							
Exclusive breastfeeding four months (binary)			X				*NCHR*
Exclusive breastfeeding (continuous)	X	X	X				*NCHR*
**Secondary**							
Readmission of infant to hospital related to nutrition	X	X					*NPR*
Experienced breastfeeding problems	X	X	X				
Family’s relationship with health visitor	X	X	X			X	
Maternal breastfeeding self-efficacy	X	X	X				
EQ-5 D^*^		X	X				
Partial breastfeeding				X	X		
**Covariates**							
Age	X					X	*SD*
Education	X					X	*SD*
Occupation	X					X	*SD*
Civil status	X					X	*SD*
Smoking	X					X	
BMI^**^	X					X	
Details about the birth (date, mode, infant’s weight)	X						*BR*
Attitude towards breastfeeding	X					X	
Previous breastfeeding experience	X						
Breastfeeding knowledge	X					X	
Sense of support from partner	X						
Assessment of support from health visitor	X					X	

*Abbreviations:* *BMI* Body Mass Index, *BR* The Birth Register, *NCHR* The National Child Health Register *NPR* The National Patient Register, *PP* postpartum, *SD* Statistics Denmark

^*^European Quality of life – 5 Dimensions (EQ-5D)

^**^Calculated using self-reported weight and height

### Statistical methods

We use random allocation to remove any influence of both measured and unmeasured variables on treatment allocation. Before conducting regression analyses, descriptive statistics will be applied to assess selection into the trial, baseline equivalence of trial arms and loss to follow-up.

Regression analyses will be applied when analysing primary outcomes. We will account for the clustered nature of data by using random effects models for binary and continuous outcomes [51] and shared frailty models for analysing time-to-event and recurrent events in continuous outcomes [52]. Intention-to-treat analyses will be conducted. These analyses will include all mother-infant dyads in the relevant trial arms irrespective of their compliance or loss to follow-up. Complete-case analyses will be conducted restricted to observations with available outcome measures [53]. For the intention-to-treat analyses, the survey data will be linked to the Danish national registers including all births; and missing outcome data will be handled by inverse probability weighting and missing covariates by multiple imputation from the registers. The probability weights will be estimated by conducting logistic regressions with outcome data (yes vs. no) as dependent variable and the following baseline variables as predictors: maternal educational, maternal age, cohabitation status, partner’s education, household income, occupational status, maternal smoking status, maternal BMI, and maternal as well as paternal psychiatric histories.

The effect of the intensified intervention will be examined by including an interaction term between treatment arms and a binary indicator of the mother being young or having low educational attainment.

All models will be analysed with treatment alone and adjusted for known predictors for breastfeeding duration collected in the survey (smoking status, maternal age, maternal education, parity, mode of delivery, maternal BMI and cohabitation status) and for design variables used in the randomisation (region of Denmark and number of annual births (< 900 births/year vs. ≥ 900 births/year). As the randomisation will hopefully have distributed the characteristics evenly between trial arms, these adjustments are made to increase the robustness of the effect estimations. Missing predictors will be imputed using multiple imputations and the results compared to the complete case analysis.

To validate the robustness of the results, the following sensitivity analyses will be performed: First, data will be restricted to mothers having received the full intervention, i.e., including pregnancy visits (June 17 and onwards), to assess the effect of receiving the full intervention compared to mothers having received the intervention without the pregnancy visits (April 26 to June 16); second, observations with no records in registries, most likely an indicator of recent immigration, will be excluded to test whether the effect of the intervention is higher among individuals familiar with the Danish healthcare system than among those who are not familiar with the system; and, third, the worst case scenario assigning no breastfeeding at four months postpartum to all individuals with missing registrations will be used to gauge the minimum effect of the treatment.

The Danish nationwide registries will further allow detailed assessment of selection into the trial (Table 3). Selection into the trial will be examined using register data from 2022, again using inverse probability weighting to assess potential selection effects.

### Multidisciplinary evaluation data

#### Difference-in-Difference design using register data

In the National Child Health Register, municipalities have been required to report data on exclusive breastfeeding since 2011. Difference-in-difference analyses will be applied to estimate the difference in change in proportions breastfeeding at four months, and in average number of weeks of breastfeeding from before (≤ 2021) to after (≥ 2022) the intervention period comparing the intervention and control arm. The advantage of this approach is that it will provide an estimate of the effect if treatment is upscaled to the entire country. The effect of the intensified intervention in the difference-in-difference analyses will be examined by including an interaction term like in the survey analyses. Data from national Danish registers will be retrieved on all mother–child dyads in the included 21 municipalities from 2015 to 2025. This part of the evaluation awaits data availability in the registries which is expected in early 2025 at the earliest. Table 3 provides information on the registers used.

#### Process evaluation

The implementation of the intervention will be analysed by process evaluation. The analysis will focus on barriers to and facilitators of the intervention, and on the dose, delivery and fidelity of the delivered intervention. Data utilised for this purpose will be quantitative and qualitative. Quantitative data will include survey data from health visitors, the health visitor records and a survey of organisational structures; and qualitative data will include interviews with families (*n* = 10), online focus group discussions with health visitors (*n* = 3), observations of breastfeeding support in the intervention clusters (*n* = 2–3), and dialogue meetings held with health visitor representatives from intervention clusters (*n* = 8).

Data from health visitors’ survey and the organisational survey will help us gain insight into any potential contamination between trial arms carried by health visitors changing employment from intervention clusters to control clusters.

#### Realist evaluation

A realist evaluation will be conducted to explore how the intervention works. Thorough analysis of the mechanisms that the intervention activates to produce the desired outcomes in the families of the intervention group will be conducted. The evaluation will highlight what works for whom under which circumstances. Qualitative data produced in individual interviews with parents (*n* = 25), observations of breastfeeding support (*n* = 30), focus group discussions with health visitors (*n* = 6) and field notes from dialogue meetings with health visitors (*n* = 10 +) will be analysed to identify the relationship between context, mechanisms and outcomes (so-called CMO configurations) [54]. We will conduct realist interviews to examine our programme theory and the causal assumptions on which it is rooted.

#### Health economic assessment

The health economic evaluation will shed light on the total health economic consequences of the intervention, including the intensified intervention. The health economic evaluation will be conducted as a cost-effectiveness analysis measuring health benefits as changes in the proportion of women breastfeeding at four and/or six months postpartum; and as a cost-utility analysis measuring the mothers’ gained quality-adjusted life years (QALYs). We measure QALYs using the widely used EQ-5D survey. QALYs include considerations related to both physical and mental well-being [55].

In both analyses, we assess two types of costs. First, direct costs related to the intervention will be assessed. Here, we distinguish between costs associated with the adaptation of the intervention and costs associated with its operation. Adaptation costs are fixed costs that are unlikely to be incurred by potential future adopters, whereas operating costs are repeated which is why separate reporting of cost types is relevant. Second, we will estimate the indirect costs (or savings) of the intervention. Like for indirect costs, we estimate the impact of the intervention on subjects’ healthcare usage in the primary and secondary healthcare sector. Such usage includes GP services and hospital admissions for both mother and child. We will consider the longest possible follow-up period. Both when considering breastfeeding and QALYs, we report the incremental effectiveness ratio (ICER). The ICER will establish the cost required to increase the share of breastfeeding mothers by a specific percentage and the cost accrued per gained QALY. Summarizing the cost-effectiveness of the intervention using ICERs allows for comparison with other healthcare interventions. Additionally, we will conduct a series of subgroup analyses to evaluate if the cost-effectiveness of the intervention depends on family characteristics.

### Patient and public involvement

The intervention was developed based on a previous successful study [27] and on a needs assessment [30] in a co-creation design with one to two health visitor representatives from each of the intervention clusters. The development was conducted in an iterative manner where health visitors tested aspects of the intervention in their real-world setting after having attended each meeting. The intervention was developed within a two-step approach: first, the contents and major outlines of the intervention were developed (January 2021 – April 2021). Next, co-creation with health visitor representatives from the intervention clusters was conducted following the described iterations (April 2021 – December 2021).

To be able to reduce social inequality in breastfeeding, it is important to investigate how the Breastfeeding Trial works in families who carry a high risk of early breastfeeding cessation, i.e., conceptualised in this study as families in which the mother is young or has a low educational attainment. These characteristics are in many ways stigmatised in high-income settings like Denmark [56, 57]. Therefore, it is essential that the intervention supports relationship built on trust and provides a sense of security between the health visitor and the family. In an effort to avoid this stigma, recruitment videos developed and employed as part of the intensified intervention were tested among mothers in the target group and revised according to their feedback. Additionally, parents in the risk group participated during the questionnaire development phase with testing of questionnaires, which generated valuable input for the process.

A reference group of parent organisations, practitioners and decision makers will be established in order to contribute knowledge and experience, qualifying the project and in the long term facilitating and ensuring the dissemination of the project and ensuring that the knowledge produced will be targeted the audience including relevant decision makers.

During monthly dialogue meetings, health visitors will have the opportunity to disclose information about any harms or adverse effects. As the intervention aims to strengthen existing standard care, it is not expected to induce harm to the study population, and no data monitoring committee has been established.

### Dissemination

The findings from the study will be reported in international peer-reviewed scientific journals and at national and international conferences. Moreover, participating municipalities, funding bodies, state and local governments and other stakeholders will be invited to participate in a national conference where the study findings will be presented. The Vancouver authorship guidelines will be adhered to and the appropriate CONSORT reporting guideline will be used when reporting the study findings.

ClinicalTrials.gov registration: NCT05311631.

## Discussion

This article outlines a cluster-randomised trial designed to increase breastfeeding duration and reduce social inequality in breastfeeding among Danish women – a population with a high uptake and long duration of breastfeeding compared with high-income countries around the world [4]. Results from Cochrane reviews have shown that healthcare services’ supportive practices may increase the duration of exclusive breastfeeding [2] which may be even further prolonged by heightening the frequency of the support offered to families in the early postpartum period [23] when cessation rates peak [58]. Occasionally, providing equal opportunities for all will not render equal outcomes. Therefore, the present trial adopts an equity approach in which those with higher risks are offered a higher dose of the intervention.

The MRC framework for complex intervention recommends that researchers go beyond investigating whether an intervention works by asking broader research questions identifying other impacts it might have, how the intervention works taking into account the context, and also considering how emerging evidence may be used to support decision making [32]. As such, the evaluation of a large intervention like the present study may benefit from taking a multiple methods stance. Besides applying an effectiveness approach, a realist evaluation will provide knowledge about how the intervention works, for whom it works and under what circumstances [36]. Furthermore, a health economic analysis will provide knowledge about whether the intervention is cost effective compared to other breastfeeding promotion interventions as well as other early-life intervention and other health interventions in general.

A trial is only as good as methods and design applied. Therefore, we have carefully considered our choices and based them on current knowledge and MRC recommendations [32]. To achieve as high as possible a response rate in the survey conducted among parents, questionnaire items have been carefully chosen based on the proposed mechanisms of change displayed in the programme theory (Fig. 2) and we made sure to minimise the length of the questionnaire. To reduce the risk of selection bias, only few exclusion criteria were applied; i.e., we applied only those criteria that contributed to elucidate differences in the likelihood of succeeding with breastfeeding or that were a safety hazard to the infant. In this way, we may have strengthened the generalisability of the findings. Moreover, the contents of the support might vary according to context, be it organizational or personal, e.g., the skills of the individual health visitors.

One strength of this study is that data will be collected in two regions of Denmark that have some of the lowest breastfeeding rates and in 21 different municipalities. This will reduce the risk of recruitment bias and allow comparisons by context of outcomes and processes, thereby extending generalisability to other settings. Another strength is that data on individuals lost to follow-up will be retrieved from registers, providing the opportunity to analyse important differences between individuals retained in the study and those who dropped out. Linkage to register data will provide further possibility to assess recruitment bias as we will identify all women assessed versus those not assessed for eligibility [51, 59].

A potential confounder of the results is that health visitors may shift employment between municipalities in the two trial arms and that mothers in the intervention clusters might have relations with mothers in control clusters, all of which may potentially cause some contamination by relaying breastfeeding-related information from the intervention group to mothers in the control group. Furthermore, the website used constitutes a specific risk as it is password protected by a generic, easily shared code only. The role of these potential risks of bias will be assessed. Furthermore, the design of the study does not allow for concealment of allocation group.

## Supplementary Information

**Additional file 1. **Visitoutline including timing of phone calls in the intensified intervention.

## Data Availability

Not applicable.

## Footnotes

1 The inclusion criteria concerning discharge from the hospital after birth was initially less than 72 h after giving birth. However, due to a lower than expected recruitment rate to the study in all project municipalities, from 18 January 2023 onwards this inclusion criteria was extended to include women discharged within the first seven days after giving birth. Simultaneously, we began to collect information about number of days admitted to the hospital after giving birth in the first maternal questionnaire distributed approximately one week postpartum, allowing us to conduct a sensitivity analysis excluding women exceeding an admittance for 72 h postpartum.
