# Supplementary material for: Effectiveness of a community-based support programme to reduce social inequality in exclusive breastfeeding: study protocol for a cluster-randomised trial
Source: BMC Public Health. 2023 Mar 8;23:450. doi: 10.1186/s12889-023-15256-z (PMC9993656; doi:10.1186/s12889-023-15256-z)
Supplement: Supplementary file 1 — Additional file 1. Visitoutline including timing of phone calls in the intensified intervention. [file 12889_2023_15256_MOESM1_ESM.docx]

**Additional File 1** | Visit outline including timing of phone calls in the intensified intervention

| **Time point (postpartum)** | Visits in the intervention arm (all mothers) | | | Phone calls for mothers in the intensified intervention group (extra for this group) | | |
| --- | --- | --- | --- | --- | --- | --- |
| Pregnancy visit approx. GA 30-32 |  |  |  |  |  |  |
| Phone call after birth |  | *Phone call* |  |  |  |  |
| 4-5 days |  |  |  |  |  |  |
| Within the first 14 days |  |  |  |  |  |  |
| *Week 2* |  |  |  |  |  |  |
| *Week 3* |  |  |  |  |  |  |
| Second half of first month |  |  |  |  |  |  |
| *Week 5* |  |  |  |  |  |  |
| *Week 7* |  |  |  |  |  |  |
| 2 months |  |  |  |  |  |  |
| *Week 9* |  |  |  |  |  |  |
| *Week 12* |  |  |  |  |  |  |
| *3½-4 months* |  |  |  |  | *Visit*** |  |
| 4 months |  | *Phone call** |  |  |  |  |
| *4½-5 months* |  |  |  |  |  |  |
| 5-6 months |  |  |  |  |  |  |

Abbreviations: Approx.: Approximately; GA: Gestational age; PP: Postpartum.
*The contact at four months postpartum in standard care practice is a visit at which the health visitor discusses introducing solids into the infant’s diet. This is changed in the intervention as to refrain from nudging parents towards introducing solids earlier than necessary.
**A visit at three and a half to four months postpartum is planned for mothers in the intensified group to provide face-to-face support between the visits held at two and five to six months. This is based on an assessment that these mothers require additional assistance.
